# INTERRATER RELIABILITY OF AN ADVANCED NONCONTACT WOUND MEASUREMENT SYSTEM USING ARTIFICIAL INTELLIGENCE

**DOI:** 10.1093/geroni/igac059.1658

**Published:** 2022-12-20

**Authors:** Jodi McDaniel, Alai Tan, Narasimham Parinandi

**Affiliations:** Ohio State University College of Nursing, Columbus, Ohio, United States; The Ohio State University College of Nursing, Columbus, Ohio, United States; The Ohio State University College of Medicine, Columbus, Ohio, United States

## Abstract

Venous leg ulcers (VLU) are recurring, disabling wounds that develop primarily in older adults with comorbidities. In an ongoing clinical trial testing a nutritional intervention researchers use advanced technology (Swift Skin and Wound) to measure VLU healing over time. This noncontact wound measurement system uses artificial intelligence to automatically calculate wound measurements and track healing progress. However, a subjective component of the Swift system involves delineating wound perimeters on photographs captured with a tablet or smartphone. To evaluate interrater reliability of the system in the current study, measurements of 11 wounds by two independent raters were assessed using an intraclass correlation coefficient (ICC). ICC estimates and their 95% confidence intervals were calculated using SPSS statistical package version 27 based on a mean-rating (k = 2), consistency, 2-way mixed-effects model. For area measures, ICC = 0.99 with 95% confidence interval = 0.998-1.0, indicating the Swift measurement system has excellent interrater reliability.

